# Genetic islands of *Streptococcus agalactiae *strains NEM316 and 2603VR and their presence in other Group B Streptococcal strains

**DOI:** 10.1186/1471-2180-5-31

**Published:** 2005-05-24

**Authors:** Mark A Herbert, Catriona JE Beveridge, David McCormick, Emmelien Aten, Nicola Jones, Lori AS Snyder, Nigel J Saunders

**Affiliations:** 1 University Departments of Paediatrics, John Radcliffe Hospital, Headington, Oxford, OX3 9DU, UK; 2 Department of Microbiology, John Radcliffe Hospital, Headington, Oxford, OX3 9DU, UK; 3 Bacterial Pathogenesis and Functional Genomics Group, The Sir William Dunn School of Pathology, University of Oxford, South Parks Rd, Oxford, OX1 3RE, UK

## Abstract

**Background:**

*Streptococcus agalactiae *(Group B Streptococcus; GBS) is a major contributor to obstetric and neonatal bacterial sepsis. Serotype III strains cause the majority of late-onset sepsis and meningitis in babies, and thus appear to have an enhanced invasive capacity compared with the other serotypes that cause disease predominantly in immunocompromised pregnant women. We compared the serotype III and V whole genome sequences, strains NEM316 and 2603VR respectively, in an attempt to identify genetic attributes of strain NEM316 that might explain the propensity of strain NEM316 to cause late-onset disease in babies. Fourteen putative pathogenicity islands were described in the strain NEM316 whole genome sequence. Using PCR- and targeted microarray- strategies, the presence of these islands were assessed in a diverse strain collection including 18 colonizing isolates from healthy pregnant women, and 13 and 8 invasive isolates from infants with early- and late-onset sepsis, respectively.

**Results:**

Side-by-side comparison of the strain NEM316 and strain 2603VR genomes revealed that they are extremely similar, with the only major difference being the capsulation loci and mobile genetic elements. PCR and Comparative Genome Hybridization (CGH) were used to define the presence of each island in 39 GBS isolates. Only islands I, VI, XII, and possibly X, met criteria of a true pathogenicity island, but no significant correlation was found between the presence of any of the fourteen islands and whether the strains were invasive or colonizing. Possible associations were seen between the presence of island VI and late-onset sepsis, and island X and early-onset sepsis, which warrant further investigation.

**Conclusion:**

The NEM316 and 2603VR strains are remarkable in that their whole genome sequences are so similar, suggesting that the capsulation loci or other genetic differences, such as pathogenicity islands, are the main determinants of the propensity of serotype III strains to cause late-onset disease. This study supports the notion that GBS strain NEM316 has four putative pathogenicity islands, but none is absolutely necessary for disease causation, whether early- or late-onset sepsis. Mobile genetic elements are a common feature of GBS isolates, with each strain having its own peculiar burden of transposons, phages, integrases and integrated plasmids. The majority of these are unlikely to influence the disease capacity of an isolate. Serotype associated disease phenotypes may thus be solely related to differences in the capsulation loci.

## Background

*Streptococcus agalactiae *(Group B Streptococcus, GBS) is a Gram positive, facultative anaerobic bacterium that is the most common cause of neonatal and obstetric sepsis, and is an increasingly important cause of septicaemia in elderly and immunocompromised patients [1]. Serotype III GBS causes approximately 37% of early-onset and 67% of late-onset neonatal GBS sepsis (compared with 13% and 5%, respectively, caused by serotype V), and is the predominant serotype causing late-onset meningitis [1,2]. Serotype V prevails in invasive infection in non-pregnant adults (causing 29% of all such infections) [3]. The genetic determinants of the propensity of serotype III GBS to cause late-onset sepsis and meningitis have not been fully elucidated, but the availability of whole genome sequences of a serotype III isolate (strain NEM316) and a serotype V isolate (strain 2306VR) brings this prospect closer [4,5]. One possibility is that the serotype III GBS has pathogenicity islands (PAIs) that are not present in the other serotypes, and which confer an enhanced invasive potential. Glaser et al. [4] described fourteen regions of strain NEM316 that they considered to be putative PAIs. These islands are composed of 11 to 77 genes and contain most of the mobile elements in the NEM316 genome [4]. Six of the islands are adjacent to tRNA genes, a feature of pathogenicity islands [6], and many known or putative virulence genes of GBS are contained within these regions. For instance, *alp2 *[7] is in 'island IV', the *cyl *operon [8] is in 'island VI', and *lmb *and *scpB *are in 'island XII' [9]. PAIs are defined by the following criteria: (1) they carry one or more virulence genes, (2) they are present in the genome of pathogenic bacterium but absent in non-pathogenic representatives of the same species, (3) they are frequently located adjacent to tRNA genes, (4) they are associated with mobile genetic elements and are often flanked by direct repeats (DR), (5) they are unstable and either the whole of the PAI or part of it may be deleted, and (6) often represent mosaic like structures rather than homogenous segments of horizontally acquired DNA [10].

We used the *C. elegans *database genome sequence graphical interface (AceDB) [11,12] to compare the strain NEM316 and the strain 2603VR genome sequences to identify serotype III and V genomic differences, and to further define the putative PAIs in the NEM316 serotype III strain. We then conducted PCR amplification and targeted microarray-based comparative genome hybridization (CGH) studies aimed at delineating the nature of the putative PAIs.

## Results

### NCBI and AceDB analysis of the sequenced serotype III and V genomes

**Figure 1:**
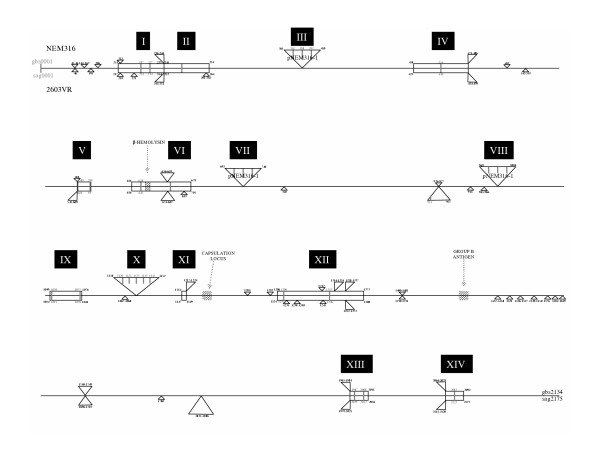
**A representation of the serotype III (NEM316; gbs001-2136) and serotype V (2603VR; sag001-2175) genomes (diagrammatic and not to scale)**. The genome sequences are mostly identical (represented by a horizontal line), triangles above the line represent gene regions unique to NEM316, and the triangles below are those present only in 2603VR. Boxed regions are putative PAIs (marked I to XIV). Grey bars with the PAIs represent genes amplified as surrogate markers for the presence of the whole island. Similar information can now be visualised through GenePlot, the NCBI pairwise comparison of protein homologs .

Side-by-side comparison of the serotype III and V genome sequences, strains NEM316 and 2603VR respectively, identified numerous annotation differences between open reading frames, most generated by true or sequencing error frame shifts and differences in the annotation of initiation codons. The similarity of the two genomes is otherwise remarkable (see figure 1). The other major differences between the two genomes are the capsulation loci and the presence of multiple mobile elements including integrated plasmids, prophages, transposons, and one to two gene integrases/transposases. Much of this acquired DNA appears to be unique to each sequenced strain (represented by triangles in figure 1), in the type of mobile element but not necessarily the genomic location.

### Which islands appear to be real PAIs?

PAIs contain virulence and mobilization genes and are flanked by direct repeat (DR) sequences that are recognised by mobilization proteins [10]. Potential PAIs must be distinguished from non-mobile regions of the chromosome that contain virulence genes adjacent to tRNA genes, and which have merely attracted mobile elements. Such mobile elements may themselves be genomic or metabolic islands but by definition they are not PAIs, unless they mobilize virulence genes and are associated with pathogenic strains [10].

Our annotation of the putative PAIs is given in table 1. The putative PAIs are present in both strain NEM316 and strain 2603VR, with the exception of islands III, VII, VIII and X, which are only present in strain NEM316. Islands III, VII, and VIII were described as identical copies of a chromosomally integrated plasmid, designated pNEM316-1. Two further islands are present in strain 2603VR that are not present in strain NEM316: sag0915-0937 (a copy of *Tn916*) and sag1835-1886 (a prophage). None of these mobile elements contain known virulence genes, and they may therefore not be true PAIs.

Inserted into the ends of islands II, IV, V, XI, XIII and XIV, and the middle of island VI, are mobile elements that contain phage or transposon genes, but no known virulence genes (see table 1). The mobile elements in strain NEM316 are different from those in strain 2603VR at each of these sites of insertion. The putative PAIs do not otherwise contain mobilization genes or flanking DR sequences. Island IX contains a two-component regulator, but has no mobilization genes. These putative PAIs may therefore merely represent non-mobile regions of the genome into which phages and transposons have inserted.

Islands I, VI and XII contain virulence genes (*rgg*[4,13,14], the *cyl *locus, and *lmb*/*scpB*[9], respectively) flanked by mobilization genes that are present in both sequenced strains. Island X contains mobilization genes and is presumed to be mobile because it is only found in strain NEM316 and not strain 2603VR. It also contains genes encoding surface proteins that have an LPXTG signal sequence; these could potentially have a virulence role. Four regions of the GBS genome (islands I, VI, X and XII) may therefore be real PAIs.

### Other genomic differences

**Figure 2:**
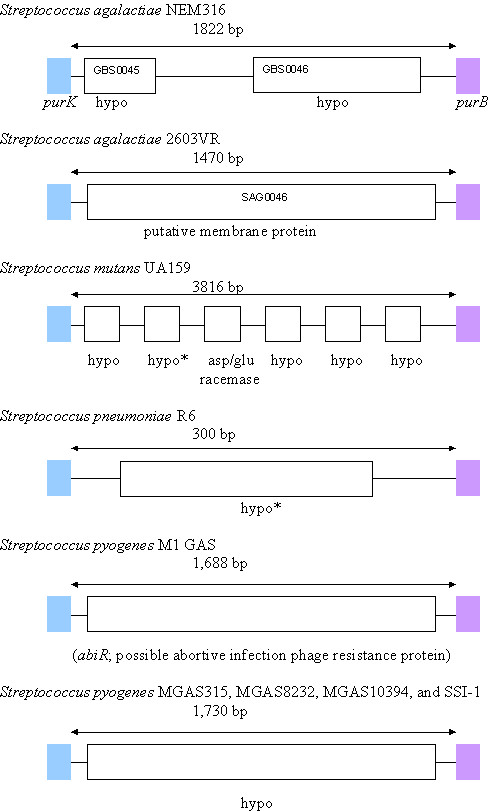
**An example of an MME in GBS**. Different intergenic regions are depicted between *purK *(pale blue block) and *purB *(lavender block) in various streptococcal species. Homologs of gbs0045 are indicated with an asterix. Hypothetical proteins are designated 'hypo'.

Aside from annotation discrepancies, mobile elements and the capsulation loci, there are few other differences between the two sequenced genomes. There is a possible lone example of a Minimal Mobile Element (MME) [15]. Two genes present between *purK *and *purB *(genes involved in purine metabolism) in strain NEM316 (gbs0045-0046) compared with a single different gene in the same location in strain 2603VR (sag0046). The putative MME was PCR amplified in each of the 39 strains in our collection. Only two insert types were amplified, of 2,036 bp and 1,636 bp. Representatives of these were sequenced and found to have the exact sequence of either gbs0045-0046 or sag0046, respectively. All strains had either one or other insert between *purK *and *purB*. Other inserts between *purK *and *purB *are identifiable in the genome sequences of other pathogenic streptococci (figure 2), hence fulfilling the criteria for an MME.

Another disparity between the two GBS whole genome sequences is the gene gbs0048 (a Cro/CI transcriptional regulator) in strain NEM316, which has a different proximal half compared to its homologue sag0048 in strain 2603VR.

### The presence of putative pathogenicity islands as defined by CGH

**Figure 3:**
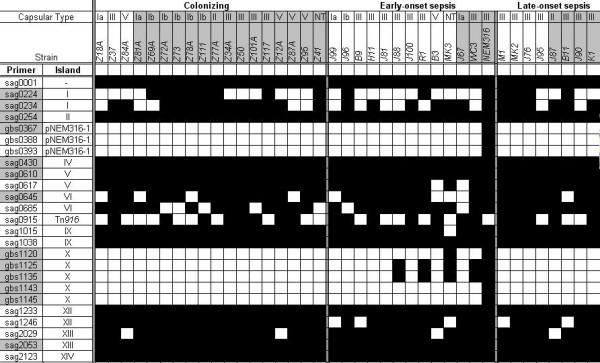
**The presence of putative pathogenicity islands as defined by PCR.**  Results of PCR (figure 3) and CGH (figure 4) analyses. The genes and GBS strains shaded grey in table 2 are those included in CGH experiments, table 3. Sag0001 encodes dnaA, a positive control for PCR. The NEM316 strain is a positive control. The pNEM316-1 plasmid is located three times in the NEM316 genome, and in figure 1 is represented as 'islands III, VII and VIII'. The strains are divided into three groups: colonizing strains from healthy pregnant women, and strains causing early- and late-onset sepsis in babies; and are sub-divided into those strains for which we have PCR results, and those for which we have PCR and CGH data.

**Figure 4:**
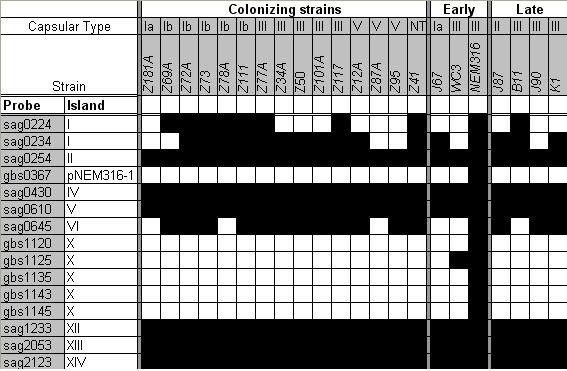
**The presence of putative pathogenicity islands as defined by CGH.**  Results of PCR (figure 3) and CGH (figure 4) analyses. The genes and GBS strains shaded grey in table 2 are those included in CGH experiments, table 3. Sag0001 encodes *dnaA*, a positive control for PCR. The NEM316 strain is a positive control. The pNEM316-1 plasmid is located three times in the NEM316 genome, and in figure 1 is represented as 'islands III, VII and VIII'. The strains are divided into three groups: colonizing strains from healthy pregnant women, and strains causing early- and late-onset sepsis in babies; and are sub-divided into those strains for which we have PCR results, and those for which we have PCR and CGH data.

**The presence of putative pathogenicity islands as defined by CGH.**  Results of PCR (figure 3) and CGH (figure 4) analyses. The genes and GBS strains shaded grey in table 2 are those included in CGH experiments, table 3. Sag0001 encodes *dnaA*, a positive control for PCR. The NEM316 strain is a positive control. The pNEM316-1 plasmid is located three times in the NEM316 genome, and in figure 1 is represented as 'islands III, VII and VIII'. The strains are divided into three groups: colonizing strains from healthy pregnant women, and strains causing early- and late-onset sepsis in babies; and are sub-divided into those strains for which we have PCR results, and those for which we have PCR and CGH data.

### Some putative PAIs are almost always present in the strain collection

Islands II, IV, V, IX and XII-XIV are almost always present in every strain from our strain collection. An occasional gene could not be amplified in one or more strains. For instance, sag1246, located in the distal half of island XII, could not be amplified in strains J99, B9, MK3, M1, and J87 (see figure 3). However, sag1233, located in the proximal half of island XII, could be amplified in all strains. The whole genome comparison of strains NEM316 and 2603VR revealed inter-strain sequence divergence at the distal end of island XII, whereas the proximal end of island XII, containing *lmb/scpB *(sag1234-1235), is highly conserved between these two strains (see table 1). Amplification of sag1233 therefore best reflects the presence of the putative PAI. Sag1233 may be particularly hard to PCR amplify because sequence divergence affects primer annealing. Similar sequence divergence between strains may also explain our inability to amplify occasional genes in islands V and XIII.

These islands are present in all strains tested, whether isolated from disease or colonizing sites and therefore do not meet the PAI definition criteria (2) and (5), above: that they should be present in pathogenic but absent from non-pathogenic strains, and they should be unstable and delete with distinct frequencies. Colonizing is not necessarily synonymous with non-pathogenic, a fact that confounds interpretation of a genetic comparison of invasive and colonizing strains.

### Some putative PAIs are almost always absent in the strain collection

Copies of pNEM316-1, represented by islands III, VII and VIII, are only found in strain NEM316, and are consistently absent from the other strains in our collection. Five genes were amplified from island X. They were only all consistently amplifiable from strain NEM316. Two or three genes from island X, however, were amplified in 4 strains other than NEM316, reflecting either the part presence of the island in these strains or marked sequence divergence. These islands, that are absent from most disease causing strains, are unlikely to be PAIs. However, the central part of island X is present in 5 strains known to have caused early-onset sepsis, and absent from all colonizing and late-onset sepsis strains.

### Some putative PAIs are variably present in the strain collection

Two genes amplified from each of islands I and VI revealed a variable presence of these islands in the strains of our collection (see figure 3). Island I is at least part-present in 14 of 18 colonizing strains (78%), 8 of 13 early-onset sepsis strains (61%), and 6 of 8 late-onset sepsis strains (75%). Although island I meets the PAI criteria of being variably present in the species, there is no relationship between the whole or part presence of the island and whether the strain was colonizing or disease causing. The two genes amplified from island I were sag0224 and sag0234. Sag0234 is close to the only recognisable virulence gene in island I, *rgg *(sag0239; homologue of a virulence regulator in *S. pyogenes*), and thus amplification of this gene reflects the presence of the most important part of the island. Sag0234 homologues are present in 13 of 18 colonizing strains (72%), in 7 of 13 early-onset sepsis strains (53%), and in 6 of 8 late-onset sepsis strains (75%). Thus, in this relatively small collection, there is no relationship between the presence of the distal half of island I and whether the strain was a colonizing or disease causing isolate.

Island VI is at least part present in all colonizing strains, 12 of 13 early-onset sepsis strains, and all late-onset sepsis strains. There is therefore no relationship between the island and disease. The proximal marker gene sag0645 is closer to the *cyl *locus (encoding the β-hemolysin, a major contributor to virulence in GBS) than the distal marker gene, sag0685, and therefore possibly better reflects the presence of a PAI that contains Tn5252 transposon genes and the *cyl *locus. Sag0645 is present in 14 of 18 colonizing strains (78%), in 9 of 13 early-onset sepsis strains (69%), and in 7 of 8 late-onset sepsis strains (87.5%). Although these differences are not statistically significant, there is a trend towards the presence of this putative PAI in late-onset sepsis strains. A larger study is required to bear out this finding.

### Comparative genomic hybridization analysis

Comparative genomic hybridisation (CGH) analysis was performed on 22 of the 39 strains assessed by PCR. These 22 strains were randomly selected and included 15 of the 18 colonizing strains, 3 of the 13 isolates that caused early-onset sepsis, and 4 of the 8 strains that caused late-onset sepsis.

For probes to the island genes, the results of CGH (figure 4) are near identical to those of PCR (figure 3), with only a few exceptions. Notable is the hybridization of strains Z50 and K1 DNA to the gbs0367 gene probe, suggesting that this gene, and therefore possibly the whole or part of pNEM316-1, is present in these strains. However, the presence of pNEM316-1 was not detected by PCR in these or any other strain except the control NEM316 strain. Thus, perhaps the gene sequence of pNEM316-1 is divergent in strains Z50 and K1 so that the primers for PCR were unable to anneal, or that CGH detected a similar gene to gbs0367. We propose that similar reasons account for the other few discrepancies that exist between the PCR and CGH results. In general, however, the CGH and PCR results are highly consistent.

Although not the main focus of this study, the presence of the other genes for which probes were included on the sub-microarray was also assessed by CGH. Eighty five percent of all the 384 probes included on the sub-microarray gave strong hybridization signals for all strains tested, indicating that at least for the region of the gene chosen for the probe design there is very little variability between the strains. However, hybridization to 15% of the probes was variable in at least three of the 22 strains tested. In most instances there was no probe hybridization, but occasionally the hybridization signal was reduced, suggesting sequence variation within the probe region. The genes with presumed sequence divergence encoded six sortases, ten proteins with an LPXTG signal sequence, two *clp *proteases, one ABC transporter and five PTS proteins, thirteen putative or known regulators, and sixteen other proteins (see table 2). Of these, several are genes with possible virulence enhancing roles (highlighted bold in table 2), including three virulence regulators *rgf *(sag1956-7) [16], a putative *rofA*-like protein (RALP, sag1463) [17] and *rogB *(sag1409) [18], two genes in the *cyl *operon (sag0662 and sag0664) [19], *cfb *(sag2043) encoding the CAMP factor [20], and *pavA *(sag1190; adherence and virulence protein A) [21], and are therefore worthy of further disease association studies. Of note, putative homologues of the major virulence regulators of *Streptococcus pyogenes *[22], such as *mga *(sag0277), *rofA/nra *(sag1356, sag1359, sag1409, and sag1463), and *rgg/ropB *(sag1490, sag2158), and all the other identifiable regulators included on the array (reviewed by Herbert et al [23]) are non-variable in their hybridization pattern, across the strain collection.

## Discussion

By combining the results of genome comparison and PCR/CGH analysis we can make the following arguments about the likelihood that each of the putative PAIs is a true PAI:

Island I may be a true PAI. It contains the virulence regulator *rgg*, which is flanked by mobilization genes, and the whole island is variably present in strains of our collection. It does not appear to be found preferentially in GBS isolates that are known to have caused disease, but the number of isolates tested in this study may be too small to tease out small contributions of a PAI to invasiveness. A confounding factor is that the colonizing isolates in our collection may have the capacity to cause disease. Thus, our colonizing and disease isolates do not simply reflect non-pathogenic and pathogenic strains, respectively. This study is not powered to identify small contributions of a putative PAI to the propensity of serotype III to cause late-onset sepsis. Only a very large study is likely to do this.

Island II is unlikely to be a true PAI. In strains NEM316 and 2603VR there are two different mobile elements inserted at the same relative genomic location into the proximal end of the island, neither of which appears to harbour virulence genes. This suggests that the proximal end of island II is a hot spot for the insertion of mobile elements. Furthermore, the distal half of island II does not appear to have mobilization machinery and is present in all the strains within our collection. Islands III, VII and VIII are near-identical copies of a chromosomally integrated plasmid, pNEM316, which contains no known virulence determinants and which is only present in strain NEM316, and is not present within other strains within our collection. Thus, this plasmid is unlikely to be a PAI.

Islands IV and V are unlikely to be PAIs for the same reason as island II. Island VI may be a true PAI as it contains the *cyl *locus adjacent to Tn5252 (present in both strains NEM316 and 2603VR), has a mosaic-like structure, and is variably present in our strain collection. We cannot show a relationship between the presence of island VI and strains causing disease, but this may be due to limitations of the power of this study. Island IX does not contain mobilization genes and is present in all strains within our collection, making it unlikely that it is a PAI. Island X is mobile, but does not contain obvious virulence determinants. The whole of island X is only found in strain NEM316, and parts of it within four other strains causing early-onset sepsis. There may thus be an association between the middle of island X, gbs1125-1135, and the capacity of an isolate to cause chorioamnionitis. Early-onset sepsis in a newborn baby reflects invasive disease in a pregnant mother, whereas the fetus is merely a vulnerable secondary host. The potential association between island X and early-onset sepsis needs a larger study for clarification. Island XI is mostly composed of a small mobile element present in strain NEM316, but not strain 2603VR, and does not contain known virulence genes.

Island XII contains mobilization and virulence genes, has a mosaic like structure, the distal end of it is variably present in strains of our collection. It could therefore be a PAI. Our study does not have the power to identify an association between the presence of the island and disease. Islands XIII and XIV are unlikely to be PAIs for the same reason as island II.

## Conclusion

The majority of late-onset meningitis, and to a lesser extent late-onset sepsis, is caused by serotype III strains. There is likely to be a bacterial genetic basis for this invasive propensity. A comparison of the whole genome sequences of a serotype III isolate, NEM316, and a serotype V isolate, 2603VR, is remarkable in the degree of similarity of the two strains, but there are some dissimilarities. These include open reading frame annotation discrepancies, genes that show sequence divergence between strains, an MME, mobile DNA, and the capsulation loci. This study contributes to our understanding of pathogenesis by further delineating the nature of mobile elements in GBS. Individual GBS isolates probably carry their own unique aliquot of horizontally acquired genetic material. Only four (islands I, VI, X and XII) of 14 putative PAIs are likely to be real PAIs, but there is no absolute association of any of these four PAIs with strains causing disease. The strongest possible disease association is with island X and early-onset sepsis.

## Methods

### Strains and culture conditions

GBS isolates were cultured overnight in Todd-Hewitt broth (Oxoid). DNA was extracted from 39 isolates of GBS (table 3): 18 colonizing strains; 13 strains derived from babies with early-onset sepsis (early-onset sepsis); and 8 strains from babies with late-onset sepsis. The control strain was NEM316 (CIP82.45, Collection de l'Institut Pasteur).

### Genome comparisons

GBS serotype III strain NEM316 and serotype V strain 2603VR genome sequences were compared through NCBI [24,25] and using AceDB [11,12], hosted by the University of Oxford Bioinformatics Centre [26]. Additional information on domains and homologies were obtained through NCBI BLAST searches [27] and the NCBI Conserved Domain Search [28].

### Molecular Methods

DNA was extracted from a 3 ml culture of each strain using spin column technology (DNAeasy; Qiagen), following the manufacturer's recommendations with the exception that lysozyme was replaced by mutanolysin (50 units per extraction) and the cell pellet was pre-incubated with this enzyme for 60 minutes at 37°C.

Double strand sequencing was conducted by the Department of Biochemistry Core Sequencing Facility, University of Oxford, using the same primers employed for the PCR using gel extracted (Qiagen) templates. Sequencing reactions used Big Dye version 3 (Applied Biosciences) and were analyzed on an ABI377 sequencer. Sequences were assembled, evaluated, and interpreted using Chromas v2.3 (Technelysium Pty Ltd) and ClustalW [29].

### PCR analysis

A standard PCR condition, *Taq *DNA polymerase (Roche) with 1.5 mM Mg^2+^, gene-specific primers (table 4) and an annealing temperature of 56°C, was established for amplifying one to five genes from each island in the NEM316 control strain, and the same PCR conditions were used to attempt amplification in the other 38 strains in our collection. The presence of a correct size amplicon was used as a surrogate marker of the presence of the whole island. When an amplicon was not obtained from a strain, the PCR was repeated with lower stringency conditions, by increasing to 2.5 mM Mg^2+ ^and decreasing the primer annealing temperature to 52°C. For all 39 strains, the gene *dnaA *(gbs0001, sag0001) was successfully PCR amplified, indicating that there were no significant PCR inhibitors in our DNA preparations. Consistent results were achieved with the PCR independently performed twice (by DM and EA). We did not attempt to amplify a gene from 'island XI' as our genome alignment and annotation clearly identified that the major part of this island was a small prophage found in NEM316 but not 2603VR. For MME amplification, primers were designed to the 3'-end of *purK *and the 5'-end of *purB*.

### Comparative genome hybridization

Fifteen gene-specific probes from within the islands were incorporated into a 384-probe GBS sub-microarray being developed to study regulatory networks in GBS (unpublished). The other probes were designed from 369 genes representing all the identifiable regulators (including homologues of *Streptococcus pyogenes *regulators such as *rofA*, *rggB*, *mga*), all the known GBS virulence factors, stress adaptation molecules, and proteins with LPXTG sorting signals, and many transporters (focussing on ABC and PTS systems). Probe regions were chosen using AceDB [11,12], so that where the gene was present in both strains, a region of greater than 300 bp region was chosen that was near identical in each of the sequenced genomes that was devoid of repetitive elements. Primers were designed using Primer3 [30], with the product size set at an optimum of 300 bp (range 150–450 bp), the primer size at 19 bp (range 17–21), the primer Tm set at 58°C (range 54–63), the primer GC% at 40 (range 30–80), and the GC clamp option set to 1. The primers were synthesised commercially (Operon). See Additional file 1 for sequence information. Amplicons were generated using DNA extracted from the sequenced serotype III strain NEM316 [4]. The printed probes were amplified from a 1:50 dilution of these products by second-round PCR using the first-round primers, once a single band of the correct size had been obtained from the first reaction, a similar single band was confirmed from the second round PCR. PCR products were checked using 96-well E-gels (Invitrogen). Probes were spotted onto Genetix amine microarray slides in Genetix amine spotting solution for amine slides using a Qarray Mini microarray printer (Genetix) using 150 micron tipped solid tungsten pins (Genetix). FluoroLink™ Cy3-dCTP and FluoroLink™ Cy5-dCTP (Amersham Pharmacia Biotech) were incorporated into 10 μg of chromosomal DNA using random hexamer primers (Invitrogen) and DNA polymerase I, Klenow fragment (Bioline, UK). Labelled DNA:DNA probe microarray hybridizations were conducted in 4x SSC, 0.2875% SDS at 65°C overnight.

Of the 384 gene-specific probes included on the array, seven probes were directed at serotype-specific capsular polysaccharide synthesis genes and were thus hybridization controls. Another five probes were directed at genes in pNEM316-1 and 'island X' that only infrequently hybridized to the DNA from the strains in the collection.

The probes were chosen in gene regions that were of low complexity, contained no repeats, and were identical according to our alignment of the NEM316 and 2603VR genomes using AceDB.

Identification of strain differences within the non-island genes was not the initial purpose of this study. However, such variation, in the context of the relative paucity of differences found in islands genes, indicates that allelic variants of the non-island genes may explain differences in strain behaviour. A larger scale project directed at these genes, using a microarray based upon a greater number of genome sequences than were available for this project, is needed to specifically investigate this type of divergence.

Probes to genes from each of islands I-XIV were included on the array, with the exception of 'islands IX and XI'. PCR analysis demonstrated that the 'island IX' region is consistently present in all strains in our collection, and our analysis did not support the notion that it contains mobile DNA. The major part of 'Island XI' is a small prophage, and we therefore expected it not to be relevant to the virulence of the organism. One probe was included to each 'island', except for two probes to 'islands I and V', and five probes to 'island X'.

## Authors' contributions

MAH did the genome comparisons, designed the primers, probes and the sub-microarray, participated and supervised the PCR and CGH experiments, and drafted the manuscript. CJBB participated in the CGH experiments and analysed the CGH data. DMcC participated in PCR and CGHs experiments, and amplified and purified the probes for the microarray. EA helped with genome comparisons and identification of array probes, and participated in PCR experiments. NJ provided the strains (prior to publication of MLST), primers and protocols. LASS established the microarray protocols, provided scientific advice, undertook gene annotations and helped with drafting the manuscript. NJS facilitated the use of AceDB, set up the microarray infrastructure, and advised on MMEs and other aspects of the work, and helped with drafting the manuscript.

## Supplementary Material

Additional File 1 submicroarray primers. File containing the sequences for the primers used for probe PCR. Click here for file

## Figures and Tables

**Table 1:** The structure of 14 putative PAIs.

Putative PAI	Position	No. of genes	Characteristics
I	gbs0211-0235	25	Island I is adjacent to tRNA-Ala, begins with a phage integrase family site-specific recombinase (gbs0211) and a Cro/CI transcriptional regulator (gbs0212), and harbors other mobilization genes. The region contains *rgg *(gbs0230), a homologue of a virulence regulator in *S. pyogenes*.
II	gbs0236-0254	19	Inserted into the proximal end of island II, adjacent to tRNA-Leu, are 9 genes in strain NEM316 (gbs0236-0244) not present in strain 2603VR, and 7 genes in strain 2603VR (sag0245-0251) not present in strain NEM316. Gbs0236-0244 consists of genes encoding a phage integrase and other phage proteins. Sag0245-0251 consists of genes encoding hypothetical proteins and a Cro/CI family regulator. Neither of these two regions harbor known virulence genes. In the remainder of island II (gbs0245-0254 or sag0252-0264), there are no other mobilization genes or known virulence genes.
III, VII, VIII	gbs0361-0410 gbs0692-0740 gbs0969-1016	50 49 48	Islands III, VII, VIII are near-identical copies of a chromosomally integrated plasmid, designated pNEM316-1.
IV	gbs0458-0482 (gbs0458-0486*)	25 (29)*	Gbs0458-0470 (or sag0423-0433) contains several transcriptional regulators, including araC family members, and the virulence factor *alp*, but does not harbor any identifiable mobilization genes. Inserted into the distal end of island IV, adjacent to tRNA-Thr, are 16 genes in strain NEM316 (gbs0471-0486) not present in strain 2603VR, and 6 genes in strain 2603VR (sag0434-0439) not present in strain NEM316. Gbs0471-0486 contains an integrase (gbs0482) and a Cro/CI transcriptional regulator (gbs0475). Sag0434-0439 contains an IS256 family transposase (sag0434), a phage family site-specific recombinase (sag0438). Neither of these elements contain known virulence genes.
V	gbs0588-0598	11	Inserted into the proximal end of island V, adjacent to tRNA-Arg, is a single gene in strain NEM316 (gbs0588; an integrase) that is not present in strain 2603VR, and 65 genes in strain 2603VR (sag0545-0609) that are not present in strain NEM316. Sag0545-0609 contains numerous prophage lambda genes. The remainder of island V (gbs0589-0598 or sag0610-0617) harbors genes encoding a cell membrane protein complex and a two-component regulator, vncSR, flanked by two transposase genes (for instance, sag0611 a degenerate transposase and sag0618 a truncated transposase). There are no genes known to be involved in virulence in island V.
VI	gbs0616-0678	63	Island VI contains the *cyl *locus (gbs0644-0655; sag0662-0673), encoding a β-hemolysin that has been shown unequivocally to be involved in virulence. The region preceding the *cyl *locus (gbs0616-0639) in strain NEM316 contains Tn5252 transposon genes, and is identical in strain 2603VR (sag0636-0657). Downstream of the *cyl *locus, in strain NEM316, there are neither mobilization genes nor other known virulence genes. In the middle of the island, three genes in strain NEM316 (gbs0656-0658; encoding a permease and hypothetical proteins) are not present in strain 2603VR, and 10 genes in strain 2603VR (sag0674-0683; protease, endopeptidase and permease genes) are not present in strain NEM316. The distal half of island VI contains genes encoding core metabolic enzymes, and does not contain mobile elements or virulence determinants.
IX	gbs1049-1076	28	Island IX contains genes with homology to those encoding a two-component regulatory system, a carbon starvation protein, and secreted proteins, but it does not contain any mobilization genes.
X	gbs1118-1153 (gbs1118-1152*)	36 (35)*	Island X appears to be mobile in that it is present in strain NEM316 but not in strain 2603VR, and it contains transferase, relaxase and some genes homologous with those in Tn5252. It also contains 3 LPXTG genes and a DNA methyltransferase. There are no known virulence genes.
XI	gbs1214-1224	11	Island XI is composed of three genes that are present in both strains NEM316 and 2603VR, and these are involved in murein hydrolase export. Eight genes in island XI are present in strain NEM316, but not in strain 2603VR. One of these is an integrase, and the element is adjacent to a tRNA gene. None of the 8 genes appears to have a role in virulence.
XII	gbs1296-1373	78	Island XII is a good candidate for a pathogenicity island. The virulence genes *lmb *(gbs1307), and *scpB *(gbs1308), encoding laminin binding protein and C5a peptidase, respectively, are at the proximal end of island XII, and are part of a large compound transposon. Upstream of *lmb/scpB*, gbs1296-1306, are five transposon (ISSdy1) or phage related genes, and downstream of *lmb/scpB*, gbs1309-1313 and gbs1338-1340, are other transposon (Tn5252) genes. In the distal half of island XII, 24 genes are present in strain NEM316 (gbs1314-1337; encoding phage and plasmid replication genes and the *lac *operon) that do not occur in strain 2603VR. In the same relative location in the genome, 20 genes (sag1253-1272; encoding heavy metal transporters) are present in strain 2603VR that do not occur in strain NEM316.
XIII	gbs1965-2011	47	Inserted into the proximal end of island XIII, adjacent to tRNA-Lys, are 20 genes in strain NEM316 (gbs1965-1984; function mostly unknown, but not obvious virulence genes) that is not present in strain 2603VR, and 47 genes in strain 2603VR (sag1979-2025; containing several phage genes) that are not present in strain NEM316. The downstream half of the island (gbs1987-2011) is identical in strains NEM316 and 2603VR and contains genes encoding the CAMP factor, two proteases, core metabolic enzymes, two transporters, and a two-component regulator. There are no mobilization genes in this half of the island.
XIV	gbs2071-2092 (gbs2064*-2092)	22 (29)*	Inserted into the proximal end of island XIV are 16 genes in strain NEM316 (gbs2064-2079; containing numerous phage genes) that are not present in strain 2603VR, and 10 genes in strain 2603VR (sag2111-2120; containing phage genes) that are not present in strain NEM316. The remainder of the island contains genes encoding 2 two-component regulators, 2 membrane proteins and enzymes involved in metabolism, but no obvious virulence or mobilization genes.

* Re-annotation of putative pathogenicity islands based upon the location of mobile DNA present in strain NEM316 but absent from strain 2603VR.

**Table 2:** Genes variably present in GBS strains, as defined by CGH analysis

**Functional category**	**Gene**
Sortases	sag0633, sag0647-8, sag0650, sag1406-7
LPXTG proteins	sag0433, sag0645-6, sag0649, sag1333, sag1404, sag1407-8, sag1462 and sag2063
*clp *proteases	sag1294 and sag1585
Transporters	sag1517, sag1998-90, sag1902 and sag1934
Regulators	sag0048, sag0124, sag0169, sag 0637, sag0644, sag1128, sag1332, sag1359, **sag1409 (*rogB*), sag1463 (encoding a RALP)**, sag1791, and **sag1956-7 (*rgf*)**
Encoding other proteins	sag0031, sag 0624, **sag0662 (*cyl *operon), sag0664 (*cyl *operon)**, sag0825, **sag1190 (*pavA*)**, sag1283, sag1417, sag1442, sag1472, sag1510, sag1558, sag1603, sag1675, sag1772, and **sag2043 (*cfb*)**

Genes highlighted in bold have a known or likely role in virulence.

**Table 3:** Strains employed for PCR and CGH analysis.

**Serotype**	**Colonizing**	**Early-onset sepsis**	**Late-onset sepsis**
**Ia**	Z18A, **Z81A**	J99, **J67**	-
**Ib**	**Z69A, Z72A, Z73, Z87A, Z111**	J96	-
**II**	**Z77A**	-	**J87**
**III**	Z73, **Z34A, Z50, Z101A, Z117**	B9, H11, J81, J88, J100, R1, **WC3, NEM316**	M1, MK2, J76, J95, **B11, J90, K1**
**V**	Z84A, **Z12A, Z87A, Z95 **	B3	-
**NT**	**Z41**	MK3	-

The strains are a subset of those used for multilocus sequence typing (MLST) of GBS. Strains indicated in bold were assessed by PCR and CGH. Non-highlighted strains were only assessed by PCR. NT = nontypeable; NEM316 = CIP82.45 (Collection de l'Institut Pasteur).

**Table 4:** Primers for PCR

**Gene assignment in strain NEM316**	**Gene assignment in strain 2603VR**	**Putative island**	**Primer Pairs**
gbs0001	sag0001	-	5'-gtagctgatagtcctggc-3' and 5'-agtccccaactaaagcgc-3'
gbs0045-46	sag0046	MME	5'-aaatgggacacgtacgg-3' and 5'-attgccgccatctcaggg-3'
gbs0217 gbs0227	sag0224 sag0234	I	5'-caagcctttaatgctcgc-3' and 5'-aactgaaattccaatcgcc-3' 5'-tcatcgcgaaaatatggag-3' and 5'-cggtcttttagaaactgtgtcc-3'
gbs0247	sag0254	II	5'-gacttatttcaagtttatgg-3' and 5'-acccttatatacgacagc-3'
gbs0367 gbs0388 gbs0393	-	pNEM316-1 (islands III, VII and VIII)	5'-atcgatttaggattcatgcc-3' and 5'-caacattcgcaaaataagcc-3' 5'-cctagatggcgtagaggcag-3' and 5'-ttgctcacagaccataagcg-3' 5'-tcacccctgagacgtttacc-3' and 5'-gatcgtaaccacggtttgct-3'
gbs0467	sag0430	IV	5'-attgatagatcttacttgcg-3' and 5'-tgatgcaatagctattggc-3'
gbs0589 gbs0598	sag0610 sag0617	V	5'-cagggtgttcaaggctacc-3' and 5'-caagcttacgcacccaag-3' 5'-ctttcctaaaacatatttgg-3' and 5'-atatggtaaaaacttaaggc-3'
gbs0628 gbs0660	sag0645 sag0685	VI	5'-tagctcagtttgcgactgg-3' and 5'-ccaacttttgcatctgctg-3' 5'-aattcttgattgatgagcg-3' and 5'-tcagctttaatcaattccc-3'
-	sag0915	Tn916	5'-aagaccaaaagtggcgaac-3' and 5'-gcctttggattcattcctg-3'
gbs1050 gbs1073	sag1015 sag1038	IX	5'-agcagttacttgatttgcc-3' and 5'-tcctgaattagctagtcgc-3' 5'-tctgcttgagataactccc-3' and 5'-caatagcagttatcaaaggg-3'
gbs1120 gbs1125 gbs1135 gbs1143 gbs1145	-	X	5'-cctagatggcgtagaggcag-3' and 5'-ttgctcacagaccataagc-3' 5'-tcgacgtgttttacggttg-3' and 5'-accgaagagatgatgacgac-3' 5'-gggccacactagaaactgc-3' and 5'-aaatccttcatcgctcctg-3' 5'-tcacccctgagacgttacc-3' and 5'-gatcgtaaccacggtttgct-3' 5'-tctctcggcgttattgtcc-3' and 5'-acaaaagcacaagcgactg-3'
gbs1306 gbs1313	sag1233 sag1246	XII	5'-ctttactggcttcacttgg-3' and 5'-gttgatacaggcattgagc-3' 5'-gattactctaccagtgagg-3' and 5'-agaatagtctgcttcaccc-3'
gbs1987 gbs2008	sag2029 sag2053	XIII	5'-ctgacaattgctttgtttcg-3' and 5'-ggctaacccaaatgtaccg-3' 5'-gctcctctgattaatgccc-3' and 5'-caagctcttgttcggttgc-3'
gbs2082	sag2123	XIV	5'-tttctgggaaaaatcagtgg-3' and 5'-ttttcccgaacaaatgatg-3'
